# Doing activism: understanding activists’ identity, practice, and the LGBT movement in Yunnan, China

**DOI:** 10.3389/fpsyg.2025.1597440

**Published:** 2025-07-23

**Authors:** Yifu Liu, Sai Tang

**Affiliations:** ^1^Department of Social Work, School of Law, Yuxi Normal University, Yuxi, China; ^2^Department of Development and Sustainability, School of Environment, Resources and Development, Asian Institute of Technology, Pathum Thani, Thailand

**Keywords:** activist, activist identity, doing activism, identity salience, LGBT movement

## Abstract

As social movement theory evolved, activist identity gained attention for playing a crucial role in the development and outcomes of social movements. This study investigates the influence of “activist identity” on the actions and behaviors of LGBT movement activists in Yunnan, China and how these contribute to the unique characteristics of the local LGBT movement. It is a qualitative study based on in-depth interviews with 20 LGBT grassroots activists in Yunnan, China. The researchers draw a parallel between West and Zimmerman’s classic theorization of “doing gender” and the findings of this study. Building upon their original conceptualization and incorporating insights from identity theory and dramaturgy, the current study narratively analyzes how the identity of the front-line activists is influenced and shaped through their everyday action. The researchers term this as “doing activism” which connotes activism as a routine accomplishment embedded in everyday interactions that allows individuals to reaffirm their self-identity as an activist and reinforce the recognition of their belonging to the LGBT movement by “others”. The “doing of activism” is both informed by and in turn creates the idea of an “ideal activist”, a status to be achieved through everyday effort. Further, the researchers argue that “doing activism” emphasizes the importance of action as a manifestation of identity and engagement in the LGBT movement. Practical outcomes in the context of the LGBT movement in Yunnan and implications for social movement field are also discussed.

## 1 Introduction

Social movements are an important means of promoting social, cultural, and policy transformations (McAdam et al., 2012; Johnston, 2017). The LGBT movement in China that aims to promote LGBT rights, has developed significantly in the past few decades, with the LGBT community gradually growing stronger and sexual minority groups gaining recognition (Kong et al., 2021; Chia, 2019). Since 1997, China’s law ended criminalizing same-sex behavior (Amendment to the Criminal Law of the People’s Republic of China, 1997). In 2001, homosexuality was removed from the list of Chinese Classification and Diagnostic Criteria of Mental Disorders - 3 (CCMD-3), completing the process of depathologization (Huang, 2018). According to the United Nations Development Programme (UNDP) (2016) report, social inclusivity in China toward LGBT people has been progressively increasing in the 21st century, empowering a growing number of young people to openly disclose their identities. Concomitantly, the visibility and participation in the national/regional LGBT movement have gradually increased, and more LGBT groups and organizations have emerged in different cities (Chia, 2019; Mavraj, 2016). They have achieved commendable results in terms of increasing legal support, social acceptance, and media publicity (Wortham, 2021).

However, despite these notable developments, Chinese LGBT movement (and community) still faces many challenges such as social discrimination and insufficient legal protection; and the realization of an inclusive society is still a long way to go (Chen et al., 2021; Kong et al., 2021). In addition, the narrative discourse of the LGBT movement remains overly uniform, failing to acknowledge the significant regional cultural differences within China. The complexities and contradictions underpinning the emergence and continuance of the LGBT social movement in China, particularly the significant regional cultural distinctiveness, therefore, requires attention to the rich contexts to study activists’ emergent identity and engagement toward visibility, community building, and goal for a more gender-inclusive and equitable society.

Theoretical attention has shifted from the grand scenes in understanding drivers of social movement (Johnston, 2017; Della Porta and Diani, 2020) to the role of identity and the internal dynamic of the movements, as well as the perspectives of individuals and activists (Jasper, 2014; Flesher Fominaya, 2010). Many such studies have recognized that activists play crucial roles as the main participants and drivers of social movements (Hinton, 2021; Ganz, 2010). The theoretical frameworks that previous scholars created in understanding the shaping of individual behavior from different perspectives provide a foundation for this study to explore the possibility of identity and social roles influencing and shaping activists’ behavior (Simon, 2008; Goffman, 2023; Berger and Luckmann, 2016).

Under such background, this study interrogates how activist identity leads to action and behavior, and in turn how action and behavior affect LGBT^1^ movement in Yunnan, China. Yunnan is a relatively active province in promoting LGBT rights under the background the social movement wave in China, with many outstanding activities, organizations, and activists emerging over the past two decades. Therefore, the researchers specifically investigate LGBT activist identity and examine how they adapt(ed) their activism to the unique local context of Yunnan. By adopting a subjective perspective of LGBT activists in Yunnan, this study challenges the simplistic view that activists are merely resources and executors of the movement (Tilly et al., 2019).

The researchers propose the term “doing activism” to capture the dynamic social construction processes observed that shapes activists’ actions. It provides a useful lens to explore how identity operates in practice, especially in relation to a gender-sexuality-linked social movement. Based on the conceptualization of “doing gender” that stresses gender is a social construct that individuals adjust their speech, behavior, and dress, etc. according to gender norms and their own/others’ perceptions of gender (West and Zimmerman, 2009; Butler, 1988), “doing activism” connotes this activism as the accomplishment embedded in everyday interactions that allows individuals to reaffirm their self-identity as an activist and reinforce the recognition by “others”. By both being informed by and creating an “ideal activist” through everyday effort, “doing activism” emphasizes the importance of action as a manifestation of identity and validated engagement in the LGBT movement. The study highlights how the actions of the grassroots activists have ultimately contributed to the effectiveness of the social movement, specifically in the three aspects of public advocacy and anti-discrimination, community building, and disease prevention and control, in the context of the LGBT movement in China.

## 2 Literature review

LGBT movement is a sub-type of social movement that holds sexual orientation and gender identity at its core. The LGBT movement in its contemporary form in China emerged later than in the West but has developed significantly over the past few decades under the background of globalization (Moreno-Tabarez et al., 2014). Influenced by the early Western LGBT movement and its discourse on equality, terms such as gay, lesbian, sexual orientation, and gender identity have gradually been accepted in China (Bao, 2018; Kong, 2016). Meanwhile transnational knowledge influenced by feminism, queer theory, and constructivism, intersecting with local culture, has led to the emergence and widespread use of uniquely Chinese terms such as tongxinglian (同性恋, initially linked to homosexuality and its legal and medical implications), kuer (酷儿, a local adaptation of the term queer), and tongzhi (同志, comrade) (Bao, 2018; Kong, 2016).

With the emergence of identities and the widespread use of the Internet, gender minority groups and organizations have begun to actively engage in promoting rights and visibility in the broader society, leading to the emergence of increasing numbers of activists (Chia, 2019; Deklerck, 2019; Lixian, 2014). These activists initially collaborated with international organizations and governments in the field of HIV prevention and treatment, subsequently using HIV epidemic resources to establish supportive communities and aid isolated LGBT individuals (Lixian, 2014; Deklerck, 2015). Their efforts gradually evolved into a constructive shift, focusing on addressing cultural, policy, and environmental survival concerns (Chia, 2019; Lixian, 2014; Deklerck, 2015). Activists are committed to increasing the visibility of the LGBT community, reducing discrimination, combating bullying, and promoting sexual education (Chia, 2019; Parkin, 2018).

The efforts and commitments made by activists in their pursuit of social justices, through LGBT movement or other social movements, are the manifestations of activism (Dumitraşcu, 2015). Activism exhibits itself in different forms through practices and behaviors of activists, and its significant role and value in social transformation have been pointed out and stressed by scholars (Shragge, 2013; Martin et al., 2007; Jin et al., 2025). However, despite the growing academic focus on the LGBT movement in China, there is a lack of systematic attention to individual activism, particularly to LGBT movement activists.

Classic social movement theory that put emphasis on factors such as organization, resources, and structure, has been criticized for its lack of understanding and attention to the individual activists involved (McAdam et al., 2012). Traditional social movement theory often prioritizes the rationality and legality of collective action, which often led to the consequence of overlooking individual factors such as emotions, identification, and motivation (Tilly et al., 2019). Previous scholars of the new social movement had noted the importance of identity and considered it a driving force behind movements (Scott, 2023). Activists are not mere actors submerged within macro-social movements, but rather, they actively shape and influence social movements and their trajectories (Della Porta and Diani, 2020). The pivotal role of activists as organizers and leaders of social movements for their agency and creativity, has also been recognized (Della Porta and Diani, 2020). Research also indicated that the effectiveness of social movements depends on activists’ strategies, actions, abilities, and identification with the movement’s goals and values (Ganz, 2010; Meyer and Tarrow, 1998). The increasing academic attention on the significance of activist role in social movement necessitated more in-depth and nuanced investigation into factors such as their identity, emotional experiences, actions, and strategies, to better comprehend the essence and significance of social movements (Hinton, 2021; Melucci, 1989).

As social movement theory further developed, activist identity therefore gained attention for playing a crucial role in the development and outcomes of social movements (Morris and Mueller, 2005; Benford and Snow, 2024). Scholars consider activist identity to be both the core and dynamic of social movements (Armstrong and Bernstein, 2008; Polletta, 2009). Blumer (1971) proposed early that social movements is a manifestation of identity politics. Activist identity both defines the boundaries of social movements (Della Porta and Diani, 2020) and determines individuals’ preparedness and intention to participate in collective action (De Weerd and Klandermans, 1999). The degree, mode, strategy, and motivation of activist’s participation are all believed to be influenced by their identity (Polletta, 2013; Melucci, 1985; Stürmer and Simon, 2004). It should be noted that although the importance of identity is widely acknowledged, scholars tend to avoid a standardized concept of activist identity because the roles and functions of activists need to be adjusted according to different social and historical contexts (Bobel, 2007). Differences in personal values and goals inevitably lead to diversity among activist identities (Shaw, 2013).

Scholars have been making efforts to understand better activist identity through their individual behaviors from different angles. For example, in identity theory, behavior is considered to be a part of one’s identity (Simon, 2008). Identity is activated in specific contexts and subsequently affects an individual’s adoption of appropriate behavior; this mechanism is known as identity salience (ibid.). In the dramaturgical theory, Goffman (2023) similarly explicates the role of external expectations, and believed that individuals adopted and adjusted actions or performances to meet others’ systematic expectations to maintain the stability and coherence of their social roles and to obtain recognition and approval (ibid.). This is the classic concept of “taking the role from others” in symbolic interactionism (Berger and Luckmann, 2016; Goffman, 2023).

In conclusion, in social movement research, scholars have recognized the importance of the activist perspective and activist identity. Expanding the activist perspective is a prevailing trend that this study endeavors to contribute, by investigating how identity influences LGBT activists’ behavior and actions and thereby promoting a contextual understanding of the Chinese LGBT movement. By linking personal identity and everyday actions of activists with the broader LGBT movement, this study provides not only practical insights for social movement practice but also expands the discussion in social movements research.

## 3 Methodology

This study utilizes life history interviews to conduct a qualitative exploration of the experiences of senior LGBT movement activists in China. The study area is the Yunnan Province where LGBT work started relatively early in China. Benefiting from the severe AIDS epidemic in the past, local government and international foundations launched proactive social initiatives (Gåsemyr, 2015; Wortham, 2021). This directly or indirectly contributed to the formation of local LGBT communities and a well - established community foundation over time (Wortham, 2024).

Fieldwork was conducted in 2021 in six different cities with native activists from nine different cities (or ethnic autonomous prefectures) in Yunnan. The study uses purposive and typical case sampling to prioritize selecting activists who have participated in the LGBT movement for over 5 years and have a clear identity awareness. To establish relationships with local activists, the researcher utilized the Internet for preliminary contact. Some of local LGBT movement activists were publicly recognized figures within the community or broader society. The researchers contacted them through publicly available means, explaining the purpose, introducing the present study, and inviting them to participate. Upon arriving in the field, life history interviews were conducted with seven activists who had previously agreed to take part in the study after initial online contact. Meanwhile, the lead researcher immersed himself deeply in the work and lives of the participants in the local city/town through field observations, without disturbing them, to understand their contexts, backgrounds, and subcultures. The lead researcher is a native of China and he used to work at an LGBT service organization in the province, giving him the advantage of native language fluency and contextual understanding of the region and research background. To ensure objectivity and neutrality, the researcher suspended preconceived personal experiences and theoretical frameworks, refraining from employing subjective data derived from personal opinions or field observations for analysis. The purpose of field observation is to enable the researcher to fully understand the respondents’ expression habits, content, and language used, avoid missing key information in interviews, and ensure that the communication with respondents could be on the same wavelength during interviews. To eliminate the boundary between the researcher and the participants, the researcher volunteered to work in the organizations where the activists belonged. During field observations, subsequent participants were identified through in-person interactions, conferences and meetings.

Face-to-face semi-structured interviews were conducted with activists who agreed to participate in the study. The interview questions were categorized into three primary types: biographical, experiential, and reflective questions. Examples included: “Could you share about your career trajectory?” “How do you perceive your identity and position within the LGBT movement?” and “What would it be like, both situationally and emotionally, if you were no longer engaged in LGBT-related work?” The researcher deliberately employed broad, non-directive questioning techniques to provide participants with ample space to articulate their unique perspectives. A comprehensive set of probing questions was prepared in advance to facilitate in-depth exploration of particularly interesting or ambiguous points emerging from participants’ narratives. Follow-up questions were selectively employed; when respondents had already provided full accounts on certain topics, no redundant probing was conducted. Concurrently, the researcher maintained particular attentiveness to capturing emergent insights that might surface spontaneously during interviews, guaranteed that the depth and authenticity of data collection would not be constrained by potential subjective limitations inherent in the interview protocol design. The interviews were initially designed as one-on-one sessions, with each session lasting between 1.5 and 2.5 h, though the duration was adjusted flexibly based on the respondent’s engagement and circumstances. Under the premise of ensuring the quality of interviews and accommodating respondents’ schedules, practically, the number of interview sessions per respondent varied from one to four. On average, each respondent spent approximately 115 min in total across 1.76 interview sessions.

The study includes interviews with twenty activists, all of whom identify themselves as activists and 18 of them are currently active at the front lines. The average years of involvement of the participants in the LGBT movement is approximately 11 years, and 14 of them work in local LGBT NGOs as full-time staff. To ensure diversity and representation, the study prioritized the inclusion of respondents from different minority groups and with different work positions (roles). Table 1 illustrates the varied domains of involvement of these activists in the LGBT community and movement in Yunnan. Further, participants in the study had diverse ethnic and religious backgrounds and speak different dialects. Table 2 presents participants’ demographic information with care to shield directly identifiable details and maintain anonymity.

**Table 1:** Working fields of participants.

No.	Nick Name	LGBT community construction and culture development	Peer support	HIV/AIDS related work	Comprehensive sexuality education	HIV positive group service	Public advocacy and discrimination reduction	Other
1	Aming	√	√	√			√	
2	Baozi	√			√		√	Artist-activism
3	Chunbi	√	√	√	√	√	√	
4	Daole	√			√		√	
5	Ejie	√	√	√	√	√	√	
6	Faran		√	√		√		
7	Gaoli		√	√		√		
8	Huxi		√	√		√		
9	Inan		√	√		√	√	
10	Jiejie	√	√	√		√	√	
11	Kuge	√	√	√	√	√	√	Community-based organization support
12	Lijie	√	√		√		√	Disability rights activism
13	Malu	√	√	√		√	√	
14	Niu	√	√	√	√	√	√	Female sex workers and AIDS orphans program
15	Ouyang	√	√		√		√	
16	Pipi	√	√	√		√		
17	Quna	√	√	√		√	√	Community-based organization support
18	Renji	√	√	√	√	√	√	Community-based organization support, injecting drug users program, former AIDS and LGBT program officer at the international foundation
19	Sunan	√	√	√		√		
20	Tutu	√	√	√		√		Community-based organization support

**Table 2:** Demographic information of participants.

No.	Nick name in study	Language	Years of working	Active LGBT activist	Self-identified gender and sexual identity	Ethnic	Religion
1	Aming	Mandarin	7	No	GAY	Han	NA
2	Baozi	Mandarin	9	Yes	Straight male	Han	NA
3	Chunbi	Mandarin	9	Yes	GAY	Han	NA
4	Daole	Mandarin	3	Yes	Queer	Han	NA
5	Ejie	Mandarin	14	Yes	Trans female	Han	NA
6	Faran	Mandarin	6	Yes	GAY	Han	NA
7	Gaoli	Mandarin	7	Yes	GAY	Han	NA
8	Huxi	Mandarin	6	Yes	GAY	Jingpo	Ethnical primitive religion
9	Inan	Mandarin	10	Yes	GAY	Han	NA
10	Jiejie	Mandarin	15	Yes	Lesbian	Han	NA
11	Kuge	Mandarin	16	Yes	GAY	Han	Christian
12	Lijie	Mandarin	9	Yes	Lesbian	Hui	Islam
13	Malu	Mandarin	6	Yes	GAY	Han	NA
14	Niu	Mandarin	13	No (active in other field)	Straight female	Han	NA
15	Ouyang	Mandarin	20	Yes	Lesbian	Han	NA
16	Pipi	Honghe dialect	13	Yes	GAY	Han	Buddhism
17	Quna	Mandarin	17	Yes	GAY	Han	NA
18	Renji	Mandarin	10	No (active in other field)	GAY	Han	NA
19	Sunan	Mandarin	11	Yes	GAY	Yi	NA
20	Tutu	Mandarin and dialect	12	Yes	GAY	Hui	Islam and Buddhism

Ethics clearance was obtained from the review board of the authors’ home institution. Informed consent was obtained from all respondents. Confidentiality was strictly enforced for interview recordings, transcripts, and participant information. The pseudonyms used in the study were randomly assigned by the researcher and are not the nicknames used by the participants in real life. Personal identifiable details are also anonymized as much as possible, including the institutional affiliations of the activists to protect privacy and confidentiality. All interviews were recorded and transcribed verbatim for more detailed textual analysis. Descriptive codes were used to analyze transcripts due to the differentiated background, language, and understanding among different respondents. The study employed inductive coding, also called open coding, as it had no fixed codebook and all data were from firsthand data collection (Saldaña, 2016). The researchers first extracted codes from the narrative data and then engaged them in dialog with the theoretical framework, thereby further ensuring the study’s objectivity and neutrality rather than forcibly applying pre-existing theories. Narrative analysis was adopted as the data analysis method, which emphasizes shared narrative conventions in accounting of human experience (Johnstone, 2016; Labov and Waletzky, 1997).

## 4 Findings

### 4.1 Doing activism: identity and action

The researchers propose the term “doing activism” to capture the symbiotic and productive relationship between everyday actions, identity construction, and the social movement participation of the activists. The data suggests there are two complementary ways in which participants’ perception of their identity as LGBT activists shaped their behavior: (1) individuals are aware of their identity as activists and consciously take actions conducive to LGBT movements and improve or modify their own behaviors toward that goal; (2) individuals modify their behaviors and actions to conform to others’ expectations of an activist’s role/behavior. Both are manifested in their routine work and day-to-day interactions with the boundary between their activist (work) identity and self increasingly blurred for some.

#### 4.1.1 Activism as routine work

The everyday actions of the LGBT movement activist are shaped by imagining and affirming their activist identity. Further, as articulated by the respondents, their work and personal life in some instances, is inextricably linked to creating/living the ideal activist image. As respondent Aming said: “*It’s not that you have to do something, it’s that you have certain requirements for yourself; you just follow this requirement, try to live up to this requirement.*” As an illustrative example here, Aming describes himself as a person who prefers to be alone and is choosy about social interaction. Yet, given his role as an activist, he tries to be sociable and interacts with people with respect and courtesy at work, which he normally is not willing to do at a personal level.

*For example, in life, if I meet a certain kind of person, I will definitely stay away… But if you meet him at work, you will still treat him patiently as a service receiver… (Aming)*

As explicated by theory, different role identities are accompanied by specific norms in society (Stets and Serpe, 2013; Burke and Tully, 1977). Note, here the norms articulated by the respondents are not rigid codes of conduct, but subjective requirements of activists on their own. For instance, activists would like to “*give oneself some rules and even form a personal professional ethic and code of conduct*” (Ouyang). Or as another articulated, “*Since you are such a person (activist), you must always consider what you are going to do, what you can do, and what you cannot do*” (Tutu).

The statements of Aming and Tutu represent the views of respondents who shaped their actions or behaviors to conform to the idealized image of an LGBT activist either by tailoring their actions to manifest the LGBT activist identity (to do what they think should be done) or alternately changing or renouncing behaviors that are not consistent with the LGBT activist identity (to not do something that they perceive should not be done by an ideal activist).

When it comes to “being an activist,” almost all respondents repeatedly stressed the dimension of “taking action.” Thus, it strongly appeared that in the consciousness of these respondents, action and identity are naturally inseparable, and the activist’s primary duty is to act. As illustrated by Baozi, “*The influence of my identity on me…. I will be more proactive than before, not only communicating with others, but also doing things, and thinking, and being more expressive*.” This congruence between identity and action (doing) is best expressed by Daole who said, “*The identity of the activist is to do activities. When you do the activity, you start to act. Or (like) writing an article… (digressing to another story)… with this kind of action, visible things, and your identity can be transmitted… (trying to explain what he said with three consecutive examples)*.”

In other words, once individuals acquire or emphasize their identity as LGBT movement activists, their sense of action was aroused, “*whether by doing activities, projects or serving the community*” (Ouyang). As Kuge expressed that once labeled as an activist, he was inspired and was willing to go all out.

Overall, the findings indicated that the participants being labeled as activists were a meaningful identity which was not subjective in nature and gave them strength. It was not a feeble/easy label, instead, it requires service and contribution to the community through actions. That means, in a mutually reinforcing circle, having an activist identity drives the individual to practice activism and vice versa.

Interestingly, given the varied roles occupied by the respondents in this study within the broader movement and service to the LGBT community, some dissenting views emerged related to the activist label and the nature of work undertaken by various individuals. One school of thought, represented by Aming here believed that “*Engaging in advocacy work can better reflect the identity of an activist (than service work)*.” Quna took a similar view, saying activists should “*Wave the flag and shout for right*.” In other words, being a vocal advocate, expressly engaging in advocacy activities, was core to being an activist. However, most respondents voiced that positions in service roles and direct engagement with members of the community (service to the community members) were equally or more aligned with their identity as activists.

Further, the commitment to action and being an activist also extends to subtle shifts in attitudes and their active attempt to “*think like an activist*” (Chunbi) after acquiring the identity. This connotes an expectation to become more receptive to differing views, viewing issues holistically, and thinking more critically about responses.

*You can see and think about many issues that you have not thought about before. It (activist identity) may not give you a lot of feedback at once, but it does affect your thinking about some issues. When you become an actor or (organization) head. (Daole)*

Daole here is recounting his transformation from a mere participant in NGO activities to the head of the organization, and his recognition that the shift was more than just about responsibility, but also as a shift in his mindset and perspective. Similar observations on the sense of responsibility and shift in perspective – with an emphasis on being willing to be more reflective and embrace more ideas were repeatedly made by other participants as well, such as Malu, “*I was actually a very selfish person; I care the feeling of myself. But this work makes me have to accept more, like different ideas, different voices and thinking about the different possibilities*.” This also comes from a sense of duty to consider their action and the impact it could have on the community they serve… “*what activists do really matters and should be done with caution*” (Niu).

In direct line with this emphasis on action and reflection, respondents envisioned that the acknowledgment, by self and others, of their activist identity improved the quality of their activism (work). To set an example, they felt the need to make positive changes in their actions to match their identity as activists. This stems from the underlying logic of the gap between themselves and the ideal activist. Since there is no standardized template for the ideal activist, participants relied more on their own understanding and subjective imagination of what the ideal activist should be like and striving to work toward this idealized self through such self-reflexive praxis.

*I wanted to provide the best (service) every time at the beginning, but now it’s more of a steady state that I do not ask to be the best, but I must reflect on my experience every time then get some real input from the community steadily and slowly… To really get things done a little bit better. (Malu)*

Another respondent, Jiejie spent a lot of time telling a story to illustrate the changes she’s undergone since she became an activist. At the end of the telling, to confirm her intent in telling the story, the researcher probed:

*Researcher: “So you mean that this judgment and determination is more indicative of who you are now as an activist.”*

*Jiejie: Yes (firmly), (activist) as it should be.* [emphasis added]

Respondents told story after story about how they believe that embracing their identity as an activist has improved the quality of their actions (activism). Although they emphasized different entry points toward these shifts, they shared two common logics. First, because they are activists, they believed they needed to possess such qualities. The other is that in their narrative, they tried the action to enhance the effectiveness of the activism and effect a positive outcome for the movement’s goals.

Finally, several activists articulated that they felt being LGBT movement activists allowed them to take bold and authentic actions. Daole vividly likened activist identity to a cloak of transparency.

*… I feel like there is something in the outer circle with a layer of clothing. Here they (the LGBT movement/community) are wearing a transparent coat. It allows you to see and do things more realistically. (Daole)*

#### 4.1.2 Self-disciplining and the idealized activist self

Although the respondents identify strongly with being an LGBT movement activist, they still routinely reported seeing themselves as far from the “ideal activist”. Apart from stressing the reflective awareness in improving their activism, it emerged that in order to better embody the role of an ideal activist, the activists changed their behaviors, revising actions that they deemed inappropriate for this role. In other words, to match their activist identity, activists actively modified, restricted, and restrained their apparently mismatched and negative behaviors like engaging in self-disciplining. This was strongly related to their imagination of the ideal activist captured in quotes such as “*to be an activist, you need to act like an activist; similarly; a leader needs to act like a leader.” (Chunbi)*

Interestingly and ironically the most common domain respondents reported self-disciplining or modifying their behavior was in relation to the expression of their sexuality and romantic engagements. One respondent said, “*It quite hinders me from hooking up (laughs)*” (Malu). Another similarly expressed,

*At least I think that when I was in Yunnan, I did not open up more and follow my heart to fall in love or something. Because there is such an identity and you seem to be a public figure. When you are in this circle, it is easy to be judged by others. I had been still very repressed in that aspect of my (sexual) needs. (Renji)*

Activists strived to curb and correct their sexual behavior and relationships, which are perceived as undesirable. The Chinese word “乱 (luan)” (similar to dissolute habit) is commonly used in the local LGBT community, referring to a variety of active and non-mainstream sexual practices, such as frequent sex, sex with multiple partners, one-night stands, group sex and so on. Many respondents considered “luan” to be a bad behavior that did not accord with activist identity. They commonly expressed concern that having a sexual relationship or dual relationship with a community member (as a partner and someone they serve) would affect the image of the movement activists and their work.

Further, activists also discussed valuing anti-discriminatory language and behaviors and striving to avoid offending the populations and different groups in the society. There was a consensus that the fight for LGBT rights should not be pursued at the expense of some LGBT subgroups and/or other minorities. The instances given by Daole typify this:

*You will (stretched to emphasize) pay much attention to your article and your wording, and whether there are some implicit sexist words (paraphrases), for example, if she is a girl, I will use the female word, and if he is a boy, I will use the male word. This is what needs to be done, and pay attention to political correctness. (Daole)*

The degree of self-restraint of activists is visibly high. This self-restraint also extends to the self-correction of everyday speech and behavior.

*For example, sometimes when I see the (social media) accounts of some of my colleagues, I want to scold them, but because I work in an LGBT organization now, I cannot scold them (very joyful laugh), so I hold back and will not leave a message. (Baozi)*

*“Something you cannot say, something you cannot do.” (Aming)*

*I might say some of the more serious swear words when I break down, but it’s basically gone now (after becoming an activist). (Daole)*

The self-disciplining on the one hand stems from considering the possibility of adverse consequences. On the other hand, they also see themselves as role models for their community members and are reminded of the burden of being acknowledged activists. This aligns with dramaturgical theory, which emphasizes the concept of presenting oneself to others (Goffman, 2023), and is also consistent with Foucault’s theory of power and surveillance (Foucault, 2012). Here it emerges that for these activists in the LGBT movement, like prisons, schools, and other institutions, creates a sense of being constantly watched and monitored (ibid.), leading these individuals to self-censor and conform to social norms and shaping their lives. Respondents’ emphasis on correct behaviors and actions can be seen as a form of impression management in the context of activism, where these activists strive to present themselves as legitimate and qualified representatives of the movement and act in accordance with their imagination of the ideal activist.

#### 4.1.3 Doing activism and everyday interactions

All the respondents talked in depth about how other people’s expectations affected their own behaviors and actions as activists. A dialectical understanding emerged from the narrative data: where the expectation of others was consistent with the will of the activists, it drove their actions toward the actualization of a higher ideal; however, when the expectations were perceived as too high or deviate from the activists’ will, they inhibited or restricted the activists’ actions.

According to identity theory, role identity is the expectation system of an individual’s behavior in society (Stets and Serpe, 2013; Burke and Reitzes, 1991). In the interviews, the respondents confirmed the role of external expectations in shaping their behaviors/identities “*others must have expectations on you …and external expectations on you must have a certain influence*” (Jiejie). Here another activist Ejie’s story is a typical one. As an openly out transgender woman with a high level of education, she is well-known in China. Due to her reputation, many foundations, international organizations, and individuals have approached her to set up a transgender organization and take on local or regional cooperation projects. This expectation has persisted throughout her career as an LGBT activist. Other activists too reported facing similar expectations in the line of their work too. Their narratives illustrate how they perceive the outside world as having expectations of what they can do and will do based on their past work and reputation. This expectation in turn further motivates the goals and behaviors of these activists.

*For example, within the scope of what I can do, as you just mentioned, he wants us to be more caring. If I think he has needs in this regard, I will definitely fulfil the needs. …I always tell the people here that I will give him my phone number. I said my phone is on standby 24 h a day. If you have any questions you want to ask, or want to chat, you can call at any time, no problem. (Gaoli)*

*If other people have high expectations for you, you will seriously prepare for something. I am like this, if… as mentioned before, you really care about other people’s evaluation of you. For example, when someone invites you to do a training session, then you will really prepare very seriously at this time. (Inan)*

All the respondents, except for one, displayed a positive attitude toward these external expectations. Although the needs of others can sometimes go far beyond the general boundaries of the activist’s responsibilities, as in Gaoli’s case, the activists still strive to satisfy them. The primary way of thinking among the respondents is to provide better services for the community. Some like Niu also articulated their interpretation of these external expectations as opportunities for self-improvement and transformation:

*I think first, how do I understand their expectations, or their criticism or something, because I think this is because others are good for you, so he gives you some advice, criticism, and guidance. People do not have to take the risk of offending you to mention things that you are not happy with, or things that are beyond your scope… These are the things that will tell me that you have a lot of deficiencies, and make you admit that you have a lot of deficiencies. Then You will want to keep learning. (Niu)*

Notably, these activists were also well aware that their capabilities have limits and that not all expectations can be met. They expressed how expectations that were perceived to be beyond their capacity created stress, apprehension, or other negative emotions for them. Such unreachable expectations were more likely to result in inaction instead of action.

*Expectation will put pressure on me, but this pressure is both good and bad. The good thing is that it may force me to go to a higher stage and achieve a higher purpose. But there are also bad things. If this pressure makes me feel so annoying, it may also overwhelm me, and then I feel that I cannot do it, so I refuse. (Ejie)*

Interestingly Ejie’s quote also illustrates that the connection between external expectations and the effect of activism is not a given, but rather a deliberate decision. Activists are acutely aware of their own abilities and determine how much they can realistically fulfil expectations.

A compelling and insightful example is provided by Baozi, who demonstrates an unwavering sense of devotion and commitment to his organization on a daily basis. As a result, he reported being more inclined to make concessions or exert more effort for the organization, illustrating his strong sense of personal commitment to the organization and cause.

*If this kind of expectation is consistent with what I want to do, it will be more effective with less effort. If it’s something I do not want to do, and I feel that I cannot push it away from the interests of the organization or anyone else under this expectation, it will sometimes be a bit embarrassing, but I will still weigh this, because this kind of identity sometimes makes me back down a bit. (Baozi)*

### 4.2 Actions of activists and the resultant effectiveness of LGBT movement

On the one hand activists in our study believed that the achievements and the changes they have created have no clear metrics to measure and are “*hard to see in the short term, relegated to history for future generations to review*” (Ouyang). However, it was also clear that these grassroots activists believed that they have “*contributed to the effectiveness of the LGBT movement*” (Jiejie) and “*promoted the sustainable development of the movement*” (Daole). In particular in the context of Yunnan and during the time span of these activists’ engagement in local LGBT movement, three areas of contribution emerged as significant in relation to their activities and contribution.

#### 4.2.1 Contributions to disease control and health of LGBT community

Globally activists have made an outstanding contribution in helping the LGBT community to fight against sexually transmitted diseases (STD), especially in relation to challenges posed by HIV/AIDS for this population (Michael et al., 2017). The same is true in Yunnan. Most respondents in the study had previously participated or are participating in HIV and STI-related work.

This study involved several activists who had been working in the community for over two decades, they provide some historical insight on the evolution and focus of the LGBT movement in this region. Almost all of them had been engaged in community work due to early LGBT community mobilization efforts that were initiated into the movement via AIDS-related programs. In all the 20 respondents, 17 had engaged with HIV/AIDS-related work. It is worth noting that this is not limited to gay male activists. For instance, Ouyang and Jiejie, self-identified lesbian, have also been long involved in community work and worked on the AIDS support hotline for gay individuals.

Overwhelmingly, they were very positive and vocal about their contribution to the community in terms of health outcomes.

*I think we have done so much AIDS work after all, at least in front of some people who are afraid of AIDS, we can make them more aware. …. When he goes out for sex, he will remember to come to me for condoms, lubricants, or he will prepare these things himself. As much as possible, he will protect himself and others. (Faran)*

*…at least because of my presence, fewer people are infected with AIDS. Definitely. (Pipi)*

The activists recognized positive changes happening in their service receivers, because of their direct actions and sustained engagement, including promoting consciousness-raising and behavioral changes activities, educating individuals on self-protection and protecting others, and promoting self-help and peer-assistance among service receivers. These changes are not limited to service provision but have a broader impact and ultimately contributed to the sense of collective efficacy in the community’s AIDS prevention and control efforts.

#### 4.2.2 Achievements in LGBT community building

Apart from HIV/AIDS-related work, respondents gave the most attention to the community-building aspect and legacy of their activist work. The long-term and sustained dedication of these activists has built up the local LGBT community and provided a solid base for the LGBT movement in the Yunnan region. Many of the respondents in this study, like Renji, Kuge, and Quna, witnessed the movement’s inception and were active in the initial mobilization and community-building efforts from the ground up. In addition to these senior activists, the rest of the respondents have also been deeply involved in community work for many years. Referring to their influence on the LGBT movement, they relate the changes in the LGBT community over the years to their achievements. One of the things they are most proud of and what they “*most want to see*” (Jiejie) is the mobilization of the local LGBT community.

*When we do community mobilization, the community is indeed mobilized. So many people and so many voices came out. And it has a good influence on the later psychological identity or the atmosphere of the whole community. (Renji)*

*Positive, for sure. In the beginning, everyone was hiding, but it got better later, much better than before, becoming more open, and much better in every way… (Sunan)*

*Let us take the small group of more than 40 people just now as an example. One day there was a friend in the group who did not bring medicine, and then he came to me for help. Then I sent a message in the group, and many friends voluntarily went to help that person find this medicine. About half hour later, that friend’s medicine has already been purchased although I did not go. This effect is real. (Malu)*

Respondents value the changes they have made to the sense of community as their contribution to the LGBT movement. They insist that community is essential to the movement, or the movement is just a vacant shell. They focus on micro-actions and their efforts to organize and unite the community which will have ripple effects in creating bonds in the community and encouraging more people to speak up and stand together with others in the movement. Respondents showcased the belief that the significance of activism lies in its ability to influence people’s consciousness and perception through actions and services. There was a sense of hope and optimism that even if an idea is not accepted temporarily, it will plant the seeds for potential changes.

#### 4.2.3 Achievements in public advocacy and discrimination reduction

Finally, the actions and work of the activists also demonstrated the growing acceptance of the LGBT community in the broader society. The data systematically reflected how eager and vocal these activists were on the importance of connecting the LGBT community and their efforts with mainstream society. Respondents directly and proudly mentioned their achievements in promoting the visibility of the community and the movement while moving toward reducing discrimination.

*For the LGBT movement, it must have a huge impact. Of course, it’s not just us doing it, on the other hand, the government’s support, and the other is the efforts of this group. As the mainstream and everyone’s recognition and attention to this area and us are getting higher and higher, slowly everyone can open their minds. (Sunan)*

*…At least you can see it in many media, at a stage when access to information is not as convenient as it is today, this is a very good influence. (Renji)*

Daole talks about “*breaking down barriers*” (between different groups), which is a key concept. When it comes to effectiveness, they all talk about “*bringing the (LGBT) movement to the masses*,” “*raising attention and visibility of the (LGBT) movement*,” and “*promoting mainstream society’s cognition of the (LGBT) community*,” and so on. This shows that these activists strongly believe that “*the LGBT movement cannot work solely within the community*” (Quna); rather, it must find a way to communicate with mainstream society to achieve effectiveness. In this context, the repeated mentioning of “*anti-discrimination*” is both an end and a means.

These local activists tend to interpret the LGBT movement more conservatively and unpretentiously. When it comes to the effectiveness of their actions and activism, they are willing to discuss how their work and efforts contribute to the community. To them, the effectiveness of the LGBT movement seems to be indistinguishable from positive change in the LGBT community and the social acceptance it brings. This is not because of a lack of knowledge about the LGBT movement or social movements. On the contrary, people like Quna, Ouyang, and Jiejie repeatedly mentioned their experiences of receiving various training and participating in LGBT movements in other countries. They would also like to talk about the differences between the Chinese and Western forms of the LGBT movement. While identifying with the policy advocacy-driven LGBT movement prevalent in the West, the interviewed activists firmly believe that a fundamental change in people’s thinking is the key to real transformation and they dedicate their efforts accordingly. In general, these local activists appeared committed to bringing effectiveness to the LGBT movement with down-to-earth and practical work.

## 5 Discussion

The findings of this research affirm that an LGBT activist identity has a positive impact on the behaviors and actions of activists, which is consistent with identity theory (Simon, 2008). The researchers draw a parallel between West and Zimmerman’s (2009) conceptualization of “doing gender” and the findings here which is termed as “doing activism”. Using the lens of “doing activism”, the study explicates how activists actively engage in everyday actions to align their self and public personas with their beliefs and the idealized image of an activist, which also reflects their commitment to the LGBT movement. Everyday actions are informed by others’ expectations and simultaneously reaffirm activist identity. These activists tailor their actions based on their imagination or the normative idea of an “ideal activist”, which they use as a guide for their own actions. The findings emphasize that activist identity plays a critical role in shaping their actions, which in turn reinforce their identity and together enhance the effectiveness and sustainability of the LGBT movement. In the context of this study, the identity construction and actions of the LGBT movement activists in the Yunnan region bring muti-faceted positive effects on LGBT movement particularly in areas such as HIV/AIDS prevention, community building, and public advocacy.

We define this bi-directional process as “doing activism”. The “doing” in “doing activism” emphasizes the importance of action as a manifestation of identity. The “activist” in “doing activism” stresses the significance of the image of the ideal activist. While the literature on the social movement field does not necessarily provide a consensual standard for the ideal activist (Shaw, 2013), individual and contextual standards for the ideal activist also vary. Yet, the study suggests that both the activists (as subject/object) and others’ gaze shape activists’ actions based on their (respective) understanding and imagination of the ideal activist. Further, the mindfulness to others’ gaze links activists’ self and other individuals. Self’s and others’ imagination of the ideal LGBT activist together constitute an idealized image, according to which the activists both self-discipline and modify their thoughts and behavior. In other words, their actions would be directionless without the imagination of the ideal activist.

Some scholars believe that the perfect activist standard will cause resistance to activist identity (Bobel, 2007; Corrigall-Brown, 2011). In line with that, the data of this study suggests that excessively high standards and “good activist” expectations from others serve as a source of stress and discouragement. Interestingly, a noteworthy phenomenon in terms of identity, action, and expectations emerged in connection with activists’ self-discipline regarding their sexual and romantic conduct. Frequently, they experienced a tension between their own behaviors and the idealized expectations placed upon activists. The simultaneous coexistence of identity’s facilitation in actions and the counterproductive effects of excessive demands on actions, demonstrates that the mechanism of identity salience (Settles, 2004; Stryker and Serpe, 1982) is not a singular positive process. This study provides a more comprehensive theoretical elaboration on its understanding. In conclusion, the researchers argue that the lens of “doing activism” is a productive conceptualization to understand the construction and maintenance of activists’ identity within dynamic social movement. It highlights an emphasis on the action-oriented, agentic philosophy imbued in the conceptualization while at the same time stressing the philosophy of working for/with the community and taking practical actions emergent, in the narratives of these activists. Additionally, this conceptualization illustrates the inter-connectedness between activists’ identity, daily practices, grassroots efforts, and broader societal change.

It cannot be ignored that this study is subject to notable perspectival limitations stemming from the demographic composition of local activists. Firstly, this research homogenizes LGBT groups by failing to differentiate how individuals with diverse sexual and gender identities enacting “doing activism.” More critically, the study primarily includes gay respondents and does not encompass bisexual and other gender minority groups. These deficiencies should be the issues directly addressed in more rigorous academic examinations in future.

## Data Availability

The original data from the study contains extensive information that could potentially identify the participants’ true identities. Due to privacy and/or publication of this article ethical considerations, data is not available. Requests to access the datasets should be directed to bestyifu@outlook.com.

## Footnotes

^1^LGBT refers to Lesbian, Gay, Bisexual, Transgender, Queer, Questioning, Intersex, and other diverse sexual and gender minorities not explicitly listed. Here, the abbreviated form LGBT (using only the four letters) is adopted based on the respondents’ usage and expressive habits, while its meaning remains consistent with the more recent and inclusive term LGBTQI+.

## References

[ref1] Amendment to the Criminal Law of the People’s Republic of China. (1997). 中华人民共和国主席令(第83号) [Order of the President of the People’s Republic of China (No. 83)]. Available online at: https://www.shui5.cn/article/0a/140138.html?u_atoken=144843e9caa17f056a30b48d81c4b1b6&u_asig=0a472f8c17525083804753563e007a (Accessed July 14, 2025).

[ref2] Armstrong E. A. Bernstein M. (2008). Culture, power, and institutions: a multi-institutional politics approach to social movements. Sociol Theory 26, 74–99. doi: 10.1111/j.1467-9558.2008.00319.x

[ref3] Bao H. (2018). Queer comrades: gay identity and Tongzhi activism in postsocialist China, vol. 217. Copenhagen: Nias Press.

[ref4] Benford R. D. Snow D. A. (2024). “Framing processes and social movements: an overview and assessment” in ed. A. Scott. New critical writings in political sociology, (London: Routledge), 109–137.

[ref5] Berger P. Luckmann T. (2016). “The social construction of reality” in Social theory re-wired. eds. Longhofer W. Winchester D. (New York: Routledge), 110–122.

[ref6] Blumer H. (1971). Social movements as collective behavior. Soc. Probl. 18, 298–306. doi: 10.2307/799797

[ref7] Bobel C. (2007). ‘I'm not an activist, though I've done a lot of it’: doing activism, being activist and the ‘perfect standard’ in a contemporary movement. Soc. Mov. Stud. 6, 147–159. doi: 10.1080/14742830701497277

[ref8] Burke P. J. Reitzes D. C. (1991). An identity theory approach to commitment. Soc. Psychol. Q. 54, 239–251. doi: 10.2307/2786653

[ref9] Burke P. J. Tully J. C. (1977). The measurement of role identity. Soc. Forces 55, 881–897. doi: 10.1093/sf/55.4.881

[ref10] Butler J. (1988). Performative acts and gender constitution: an essay in phenomenology and feminist theory. Theatr. J. 40, 519–531. doi: 10.2307/3207893

[ref11] Chen K. Cai W. Jin Y. Zhang C. (2021). Actuality of Chinese LGBT group. In: 2021 International Conference on Public Relations and Social Sciences (Amsterdam: Atlantis Press), 1062–1066. doi: 10.2991/assehr.k.211020.306

[ref12] Chia J. L. (2019). “LGBTQ rights in China: movement-building in uncertain times” in ed. S. Biddulph, H. Rosenzweig. Handbook on human rights in China (Cheltenham UK, Northampton USA: Edward Elgar Publishing), 657–680. doi: 10.4337/9781786433688.00043

[ref13] Corrigall-Brown C. (2011). Patterns of protest: Trajectories of participation in social movements. Redwood City, California: Stanford University Press.

[ref14] De Weerd M. Klandermans B. (1999). Group identification and political protest: farmers' protest in the Netherlands. Eur. J. Soc. Psychol. 29, 1073–1095. doi: 10.1002/(SICI)1099-0992(199912)29:8<1073::AID-EJSP986>3.0.CO;2-K

[ref15] Deklerck S. (2015). Queer comrades-a visual ethnographic study of activism in China's contemporary LGBT movement. [Doctoral dissertation]. Leuven: KU Leuven.

[ref16] Deklerck S. (2019). “Chinese LGBT+ activism—playing, organizing, and playful resistance” in China’s youth cultures and collective spaces. eds. Frangville V. Gaffric G. (London: Routledge), 150–169.

[ref17] Della Porta D. Diani M. (2020). Social movements: An introduction. 3rd Edn. Hoboken, NJ: Wiley-Blackwell.

[ref18] Dumitraşcu V. (2015). Social activism: theories and methods. Rev. Univ. Sociol. 11, 84–94. Available online at: https://www.ceeol.com/search/article-detail?id=716528

[ref19] Flesher Fominaya C. (2010). Collective identity in social movements: central concepts and debates. Sociol. Compass 4, 393–404. doi: 10.1111/j.1751-9020.2010.00287.x

[ref20] Foucault M. (2012). Discipline and punish: The birth of the prison. (A. Sheridan, Trans.). New York: Vintage (Original Work Published 1975).

[ref21] Ganz M. (2010). Why David sometimes wins: Leadership, organization, and strategy in the California farm worker movement. New York: Oxford University Press.

[ref22] Gåsemyr H. J. (2015). Twenty years of mobilising around AIDS in China: the main actors and influences behind organisational growth. Asian Stud. Rev. 39, 609–627. doi: 10.1080/10357823.2015.1087464

[ref23] Goffman E. (2023). “The presentation of self in everyday life” in ed. W. Longhofer, D. Winchester. Social theory re-wired (New York: Routledge), 450–459.

[ref24] Hinton E. (2021). America on fire: The untold history of police violence and black rebellion since the 1960s. New York: Liveright.

[ref54] Huang Y. (2018). Media representation of Tongxinglian in China: a case study of the people’s daily. J. Homosex. 65, 338–360. doi: 10.1080/00918369.2017.1317475, PMID: 28394731

[ref25] Jasper J. M. (2014). Protest: A cultural introduction to social movements. Cambridge: Polity Press.

[ref26] Jin J. Tang S. Liu Y. Luo X. (2025). “Stay committed on the frontlines”: sustainability of the activism of social workers in Guiyang, China. PLoS One 20:e0324828. doi: 10.1371/journal.pone.0324828, PMID: 40455845 PMC12129209

[ref27] Johnstone B. (2016). ‘Oral versions of personal experience’: Labovian narrative analysis and its uptake. J. Sociolinguistics 20, 542–560. doi: 10.1111/josl.12192

[ref28] Johnston H. (2017). What is a social movement? Jaipur: Rawat.

[ref29] Kong T. S. (2016). The sexual in Chinese sociology: homosexuality studies in contemporary China. Sociol. Rev. 64, 495–514. doi: 10.1111/1467-954X.12372

[ref30] Kong T. S. Kuan H. W. Lau S. H. Friedman S. L. (2021). “LGBT movements in Taiwan, Hong Kong” Oxford research encyclopedia of politics. doi: 10.1093/acrefore/9780190228637.013.1275

[ref31] Labov W. Waletzky J. (1997). Narrative analysis: oral versions of personal experience. J. Narrat. Life Hist. 7, 3–38. doi: 10.1075/jnlh.7.02nar

[ref32] Lixian H. H. (2014). LGBT activism in mainland China: a brief movement overview. Against the Curr. 29:19. Available online at: https://againstthecurrent.org/atc173/p4289/

[ref33] Martin D. G. Hanson S. Fontaine D. (2007). What counts as activism? The role of individuals in creating change. Womens Stud. Q. 35, 78–94. Available online at: https://www.jstor.org/stable/27649696

[ref34] Mavraj A. (2016). The LGBT movement in China: public perception, stigma, and the human rights debate. Inquiries J. 8, 1–2. Available online at: http://www.inquiriesjournal.com/a?id=1503

[ref35] McAdam D. Tarrow S. Tilly C. (2012). Dynamics of contention. Cambridge: Cambridge University Press. doi: 10.1017/CBO9780511805431

[ref36] Melucci A. (1985). The symbolic challenge of contemporary movements. Soc. Res. 52, 789–816.

[ref37] Melucci A. (1989). Nomads of the present: Social movements and individual needs in contemporary society. Philadelphia: Temple University Press.

[ref38] Meyer D. S. Tarrow S. (1998). “A movement society: contentious politics for a new century” in ed. D. S. Meyer and S. Tarrow. The social movement society: Contentious politics for a new century (Lanham, Boulder, New York, Toronto, Oxford: Rowman & Littlefield Publishers), 1–28.

[ref39] Michael P. Argüello T. M. Wilson C. (2017). “Practice with the gay male community” in Social work practice with the LGBTQ community: The intersection of history, health, mental health, and policy factors. ed. Dentato M. P. (New York: Oxford University Press), 286–314.

[ref40] Moreno-Tabarez U. Chávez K. R. Leonelli S. J. Huang A. Deklerck S. Rother C. (2014). Queer politics in China: a conversation with “Western” activists working in Beijing. QED J. GLBTQ Worldmak. 1, 109–132. doi: 10.14321/qed.1.3.0109

[ref41] Morris A. D. Mueller C. M. (2005). Frontiers in social movement theory. Beijing: Peking University Press. (Original Work Published 1992).

[ref42] Parkin S. (2018). LGBT rights-focused legal advocacy in China: the promise, and limits, of litigation. Fordham Int'l. L.J. 41:1243. Available online at: https://ir.lawnet.fordham.edu/cgi/viewcontent.cgi?article=2718&context=ilj

[ref43] Polletta F. (2009). It was like a fever: Storytelling in protest and politics. Chicago: University of Chicago Press.

[ref44] Polletta F. (2013). Participatory democracy in the new millennium. Contemp. Sociol. 42, 40–50. doi: 10.1177/0094306112468716

[ref45] Saldaña J. (2016). The coding manual for qualitative researchers. Los Angeles: Sage Publications.

[ref46] Scott A. (2023). Ideology and the new social movements. London: Routledge. (Original Work Published 1990).

[ref47] Settles I. H. (2004). When multiple identities interfere: the role of identity centrality. Personal. Soc. Psychol. Bull. 30, 487–500. doi: 10.1177/0146167203261885, PMID: 15070477

[ref48] Shaw R. (2013). The activist's handbook: Winning social change in the 21st century. Berkeley and Los Angeles: University of California Press.

[ref49] Shragge E. (2013). Activism and social change: Lessons for community organizing. Ontario: University of Toronto Press.

[ref50] Simon B. (2008). Identity in modern society: A social psychological perspective. New Jersey: Wiley-Blackwell Publishers.

[ref51] Stets J. E. Serpe R. T. (2013). “Identity theory” in Handbook of social psychology. eds. DeLamater J. Ward A. (Dordrecht: Springer), 31–60.

[ref52] Stryker S. Serpe R. T. (1982). “Commitment, identity salience, and role behavior: theory and research example” in Personality, roles, and social behavior. eds. Ickes W. Knowles E. S. (New York: Springer), 199–218.

[ref53] Stürmer S. Simon B. (2004). Collective action: towards a dual-pathway model. Eur. Rev. Soc. Psychol. 15, 59–99. doi: 10.1080/10463280340000117

[ref55] Tilly C. Castañeda E. Wood L. J. (2019). Social movements, 1768–2018. 4th Edn. New York: Routledge.

[ref56] United Nations Development Programme (UNDP) (2016). Being LGBTI in China: A national survey on social attitudes towards sexual orientation, gender identity and gender expression. Available online at: https://www.undp.org/china/publications/being-lgbti-china (Accessed July 14, 2025).

[ref57] West C. Zimmerman D. H. (2009). Accounting for doing gender. Gender Soc. 23, 112–122. doi: 10.1177/0891243208326529

[ref58] Wortham A. T. (2021). Playing gay: Organizing Tongzhi fun and HIV/Aids politics in Southwest China. [Doctoral dissertation]. New York: Columbia University. doi: 10.7916/d8-pqha-4d49

[ref59] Wortham A. T. (2024). Collusive infrapolitics: the hidden gay worlds of HIV community based organizations in Kunming, China. J. Contemp. China 33, 120–134. doi: 10.1080/10670564.2023.2223152

